# Structural Neural Correlates of Physiological Mirror Activity During Isometric Contractions of Non-Dominant Hand Muscles

**DOI:** 10.1038/s41598-018-27471-5

**Published:** 2018-06-15

**Authors:** Tom Maudrich, Rouven Kenville, Jöran Lepsien, Arno Villringer, Patrick Ragert

**Affiliations:** 1 Institute for General Kinesiology and Exercise Science, Faculty of Sport Science, University of Leipzig, Leipzig, 04109 Germany; 2 Department of Neurology, Max Planck Institute for Human Cognitive and Brain Sciences, Leipzig, 04103 Germany; 3 Clinic for Cognitive Neurology, University of Leipzig, Leipzig, 04103 Germany; 4 Berlin School of Mind and Brain, Mind and Brain Institute, Berlin, 10099 Germany

## Abstract

Mirror Activity (MA) describes involuntarily occurring muscular activity in contralateral homologous limbs during unilateral movements. This phenomenon has not only been reported in patients with neurological disorders (i.e. Mirror Movements) but has also been observed in healthy adults referred to as physiological Mirror Activity (pMA). However, despite recent hypotheses, the underlying neural mechanisms and structural correlates of pMA still remain insufficiently described. We investigated the structural correlates of pMA during isometric contractions of hand muscles with increasing force demands on a whole-brain level by means of voxel-based morphometry (VBM) and tract-based spatial statistics (TBSS). We found significant negative correlations between individual tendencies to display pMA and grey matter volume (GMV) in the right anterior cingulate cortex (ACC) as well as fractional anisotropy (FA) of white matter (WM) tracts of left precuneus (PrC) during left (non-dominant) hand contractions. No significant structural associations for contractions of the right hand were found. Here we extend previously reported functional associations between ACC/PrC and the inhibtion of intrinsically favoured mirror-symmetrical movement tendencies to an underlying structural level. We provide novel evidence that the individual structural state of higher order motor/executive areas upstream of primary/secondary motor areas might contribute to the phenomen of pMA.

## Introduction

Mirror Activity (MA) describes involuntarily occurring muscle activity in contralateral homologous limbs during unilateral movements. This phenomenon has been reported in patients with neurological disorders e.g. Parkinson’s disease^1–3^, Klippel-Feil syndrome^4^ and congenital gene mutations^5–7^. Due to the severity of the unintended contralateral muscle activity, which lead to disturbing and overt muscle contractions, these pathological forms have been termed Mirror Movements (MM). Furthermore, even in healthy humans, MA of a lesser extent has been observed which does not directly lead to involuntary movements of contralateral extremities. Yet, subliminal muscle activity of intrinsic motor units can still be detected using electromyography (EMG). Therefore, this non-pathological form is known as physiological Mirror Activity (pMA).

Generally, the performance of strictly unilateral movements of the upper limbs recquires a tightly orchestrated interplay of complex excitatory as well as inhibitory mechanisms between different cortical (primary/secondary motor) and subcortical (basal ganglia, cerebellum) areas that eventually lead to lateralized activation of the active primary motor cortex (M1) which in turn transmits the motor command to the contralateral limb through the crossed corticospinal tract^8,9^. In order to avoid involuntary activation of the inactive M1, the mechanism of interhemispheric inhibition (IHI) mediated by transcallosal fibers mostly connecting homologous brain areas is crucial to restrict the brain activation to the active M1^10^. Based on this idealized concept of unilateral motor execution, different impairments of underlying parts of the human motor system have been identified in order to explain the occurrence of involuntary MA.

Pathological forms of MA have previously been linked to uncrossed ipsilateral projections of the corticospinal tract^6,8^ which persist due to specific mutations in genes that are crucial for corticospinal axonal development and became therefore known as congenital MM^5,6^. Furthermore, abnormal spinal commisural fibers that lead to bilateral innervation of homologous motor neurons caused by bilateral transmission of the motor command to the spinal cord have been described^8,9^. Apart from these anatomical anomalies, alterations in the connectivity of subcortical brain areas, e.g. basal ganglia with secondary motor areas have been discussed, that might be able to explain overt MM in patients with Parkinson’s disease, probably caused by the gradual death of dopaminergic cells of the substantia nigra^1,11^.

In contrast to the pathological forms of MM, pMA observable in neurological healthy adults is thought to be the evolutionary remnant of the so called “basic-mirror-movement-mode” of the central nervous system (CNS)^12,13^. According to this concept, the default motor operation mode of the CNS was a mirror-symmetrical movement execution with bilateral innervations of homologous muscle groups^14,15^. Over time, an ongoing ontogenetic learning process enabled humans to decouple both hands and allowed for independent unilateral hand movements^16^. Generally, it is assumed that there is an association between the amount of pMA and the functional requirements of unilateral motor tasks. To date, pMA has been observed during the performance of simple and complex motor tasks^16^, especially during strong unimanual voluntary contractions^12,17–22^. Apart from force requirements, there are investigations reporting modulations of pMA in response to central and peripheral fatigue as a cause of repetitive exhaustive contractions^13,23–25^, increased movement frequency^16^ as well as increased cognitive load induced by simultaneously provided visual stimuli during task execution^26^.

Regarding the underlying mechanisms of pMA during strong unimanual contractions, the hypothesis of Motor Overflow has been suggested^27^. Motor Overflow is hypothesized to be the result of ongoing modulations of interhemispheric communication during unilateral contractions with progressively higher force demands, characterized by a gradual shift from predominantely interhemispheric inhibition (IHI) to interhemispheric facilitation (IHF), which in turn leads to bilateral activation of motor-relevant brain regions^12,28^. In support of this hypothesis, a structural white matter (WM) correlate, the fractional anisotropy (FA) of transcallosal fibers connecting bilateral primary motor cortices (M1) has recently been positively associated with the amount of IHF^29^ and pMA^12^ during dominant hand contractions. Besides that, there is also evidence for a contribution of secondary motor areas e.g. the right dorsal premotor cortex^11,30^ and supplementary motor area (SMA)^8,9^ modulating pMA.

Further evidence regarding the underlying neural mechanisms of pMA gained from functional magnetic resonance imaging (fMRI) points to a role of neural networks upstream of primary and secondary motor areas including the anterior cingulate cortex (ACC) and precuneus (PrC) in the precise coordination of unilateral movements^15^ and proactive movement inhibition^31^. However, it is unknown if these functional associations of higher-order executive areas are also relatable to an underlying structural level.

Here we investigated the structural neural correlates of pMA during isometric contractions of intrinsic hand muscles of the dominant and non-dominant hand by means of structural MRI (sMRI). The goal of this study was to discover associations between regional grey matter volume (GMV) as well as FA of major WM tracts and the individual tendency to display pMA in healthy right-handed adults. We aimed to provide further insights into the underlying neural structures responsible for the occurence and modulation of pMA using a whole-brain MRI analysis approach.

## Results

Participants underwent a structural magnetic resonance imaging (sMRI) session prior to behavioral testing. Immediately afterwards, they had to perform a pinch-force task outside the scanner (see section: Materials and Methods - Behavioral Experiment) with the dominant as well as the non-dominant hand while bilateral surface electromyografic (EMG) recordings of the first dorsal interosseus muscle (FDI) were acquired (see section: Material and Methods - EMG Recordings and Analysis for further details).

### Behavioral Data

As expected, side comparison of maximal isometric force during maximum voluntary contraction (MVC) testing detected a significant difference between left (non-dominant) hand and right (dominant) hand (median left hand = 84.7 N, median right hand: 97.9 N; Wilcoxon-test: z = −2.96, p = 0.003, r = 0.58).

The factor force level showed a significant effect on MVC normalized mean activities of left FDI (χ²(2) = 52.00, p < 0.001) and right FDI (χ²(2) = 52.00, p < 0.001). For pairwise post-hoc comparisons please refer to Table 1. Side comparison of voluntary mean EMG activities between left and right FDI showed no difference for all force levels (p > 0.13; r < 0.02 for all pairwise comparisons).

**Table 1:** Pairwise post-hoc Wilcoxon signed-rank tests of the mean EMG activites (20%, 50%, 80% MVC) of the voluntarily contracting FDI (z = z-value of Wilcoxon signed-rank test, p-value Dunn-Bonferroni adjusted for multiple comparisons, r = Pearson effect size, *significance).

Pairwise Comparison	Left FDI			Right FDI		
	z	p-value	r	z	p-value	r
20% MVC–50% MVC	−1.00	0.001*	0.20	−1.00	0.001*	0.20
20% MVC–80% MVC	−2.00	<0.001*	0.40	−2.00	<0.001*	0.40
50% MVC–80% MVC	−1.00	0.001*	0.20	−1.00	0.001*	0.20

**Figure 1:**
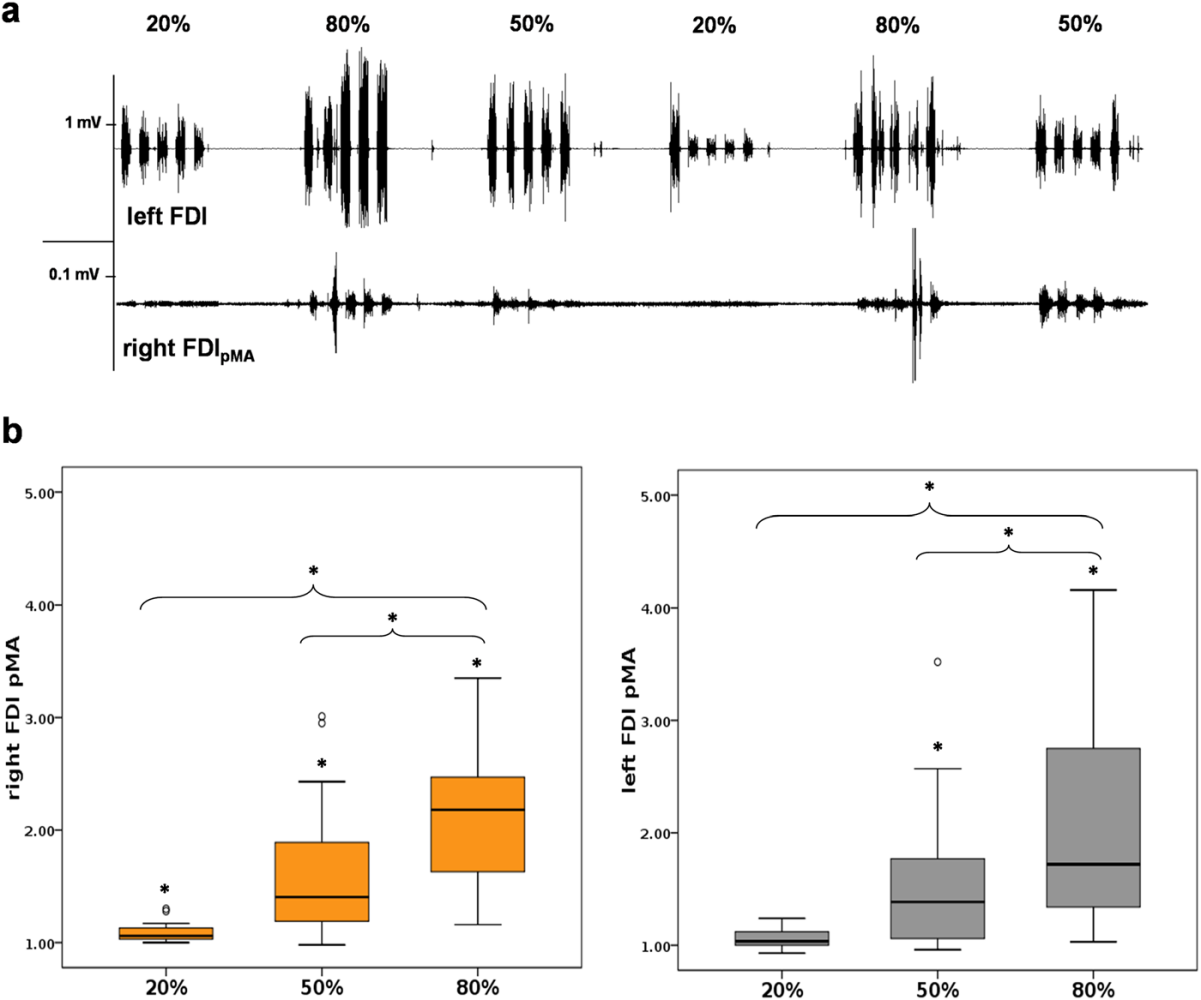
EMG-measurement and pMA values in right FDI (orange boxes) and left FDI (grey boxes). (**a**) Exemplary EMG recordings for voluntary (upper trace) and involuntary (lower trace) muscle activity. Note the different scaling of the traces and the increase in pMA depending on applied force. (**b**) All diagrams show the tested force levels (20%, 50%, 80% MVC) and involuntarily occuring mean pMA values of left and right FDI (expressed as multitudes of 1000 ms pre-burst baseline signal, value of 1 = no pMA, value of 2 = 100% increase in pMA compared to baseline activity; *indicate significant changes compared to baseline or between force levels).

With regards to pMA, the global Friedmann analysis revealed a significant effect for the factor force level on baseline normalized pMA in right FDI_pMA_ (χ²(3) = 69.30, p < 0.001) and left FDI_pMA_ (χ²(3) = 59.42, p < 0.001; see Fig. 1). For pairwise post-hoc comparisons please see Table 2. Side comparison of involuntary pMA between right and left FDI_pMA_ showed no significant differences on all force levels (p > 0.12; r < 0.30 for all pairwise comparisons).

**Table 2:** Pairwise post-hoc Wilcoxon signed-rank tests of the mean EMG activites (Baseline (BL), 20%, 50%, 80% MVC) of the involuntarily contracting FDI_pMA_ (z = z-value of Wilcoxon signed-rank test, p-value Dunn-Bonferroni adjusted for multiple comparisons, r = Pearson effect size, *significance).

Pairwise Comparison	Right FDI_pMA_			Left FDI_pMA_		
	z	p-value	r	z	p-value	r
BL – 20% MVC	−1.00	0.031*	0.20	−0.62	0.514	0.12
BL – 50% MVC	−1.85	<0.001*	0.36	−1.31	0.002*	0.26
BL – 80% MVC	−2.85	<0.001*	0.56	−2.62	<0.001*	0.51
20% MVC–50% MVC	−0.85	0.109	0.17	−0.69	0.319	0.14
20% MVC–80% MVC	−1.85	<0.001*	0.36	−2.00	<0.001*	0.39
50% MVC–80% MVC	−1.00	0.031*	0.20	−1.31	0.002*	0.26

The median slope of increasing pMA across all tested force levels (FDI_pMA20%_, FDI_pMA50%_, FDI_pMA80%_), referred to as Mirror Recruitment (MiR), was 0.0147 (0.0146) for right FDI_pMA_ and 0.0091 (0.0157) for left FDI_pMA_ (see Supplementary information, Figures S1 & S2; for an overview of individual MiR values please refer to Supplementary Information, Table S1).

### MRI Data

**Figure 2:**
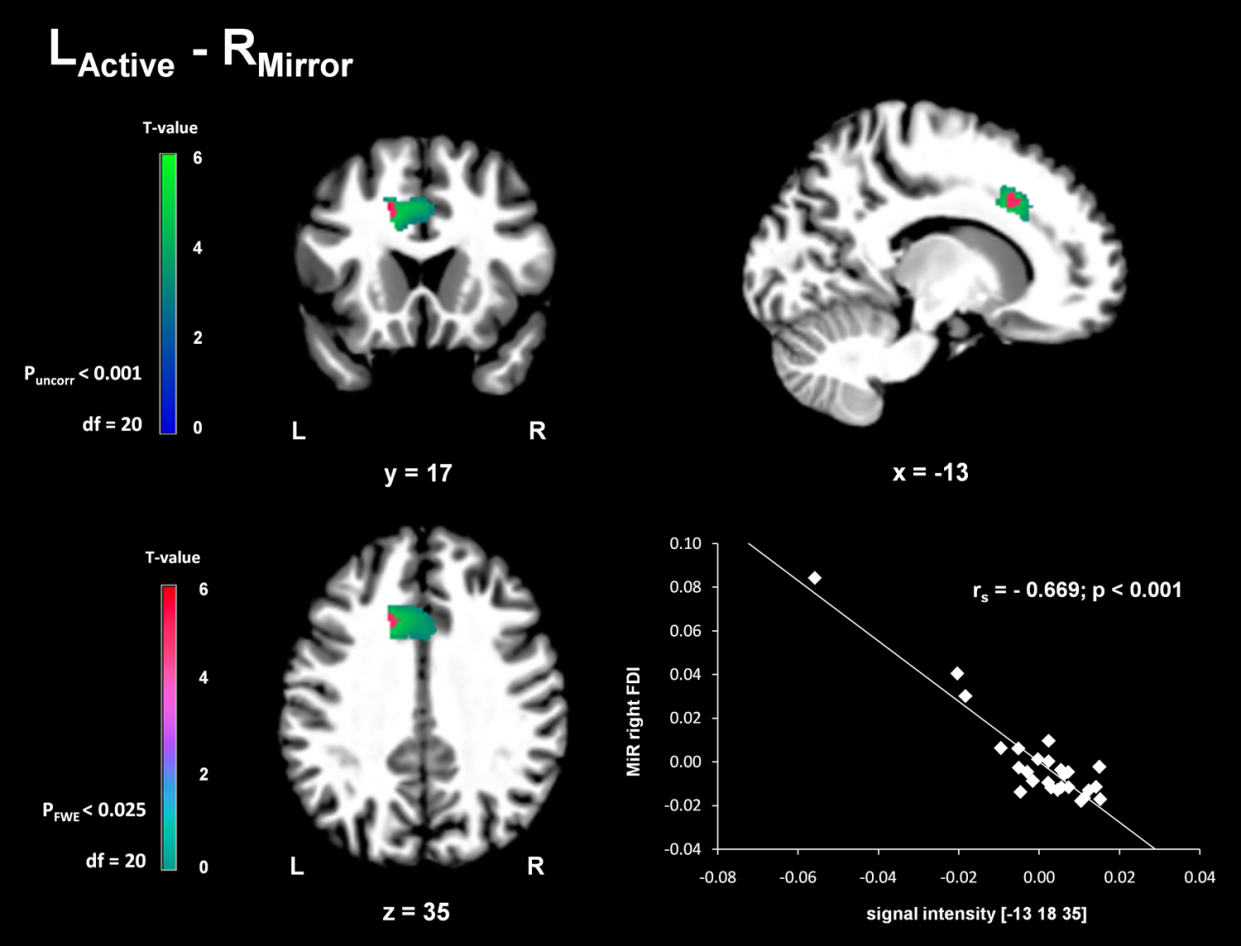
Negative correlation between GMV in left ACC and Mirror Recruitment of right FDI (R_Mirror_) during left hand contractions (L_Active_). ACC cluster (uncorrected for better visualization, p < 0.001, Center of Gravity (COG): −7/17/35; and FWE corrected, p_FWE_ < 0.025) from VBM analysis overlaid on T1 image of the most representative subject of the sample in MNI space. Scatterplot displaying the negative correlation between GMV (fitted signal intensity at −13/18/35) in left ACC and mirror recruitment (MiR) of right FDI.

VBM analysis showed that Mirror Recruitment (MiR) of the right FDI during contractions of the left FDI correlated negatively with GMV in left ACC (peak-voxel: −15/16/38; T-value = 6.33; k = 35, p_FWE_ = 0.008; see Fig. 2). Spearman rank correlation coefficient between the fitted peak-voxel signal intensity and MiR of right FDI was r_s_ = 0.669 (p < 0.001; see Fig. 2). There was no significant correlation between GMV and MiR of left FDI. However, we found a statistical trend for a negative correlation between left FDI MiR and GMV of left M1 (peak voxel: −34/−30/39; T-value = 4.89; k = 224, p_FWE_ = 0.077).

Furthermore, we tested for possible correlations between GMV and each individual force level (20%, 50% & 80% MVC). However, no significant associations for both FDI were found in these analyses.

**Figure 3:**
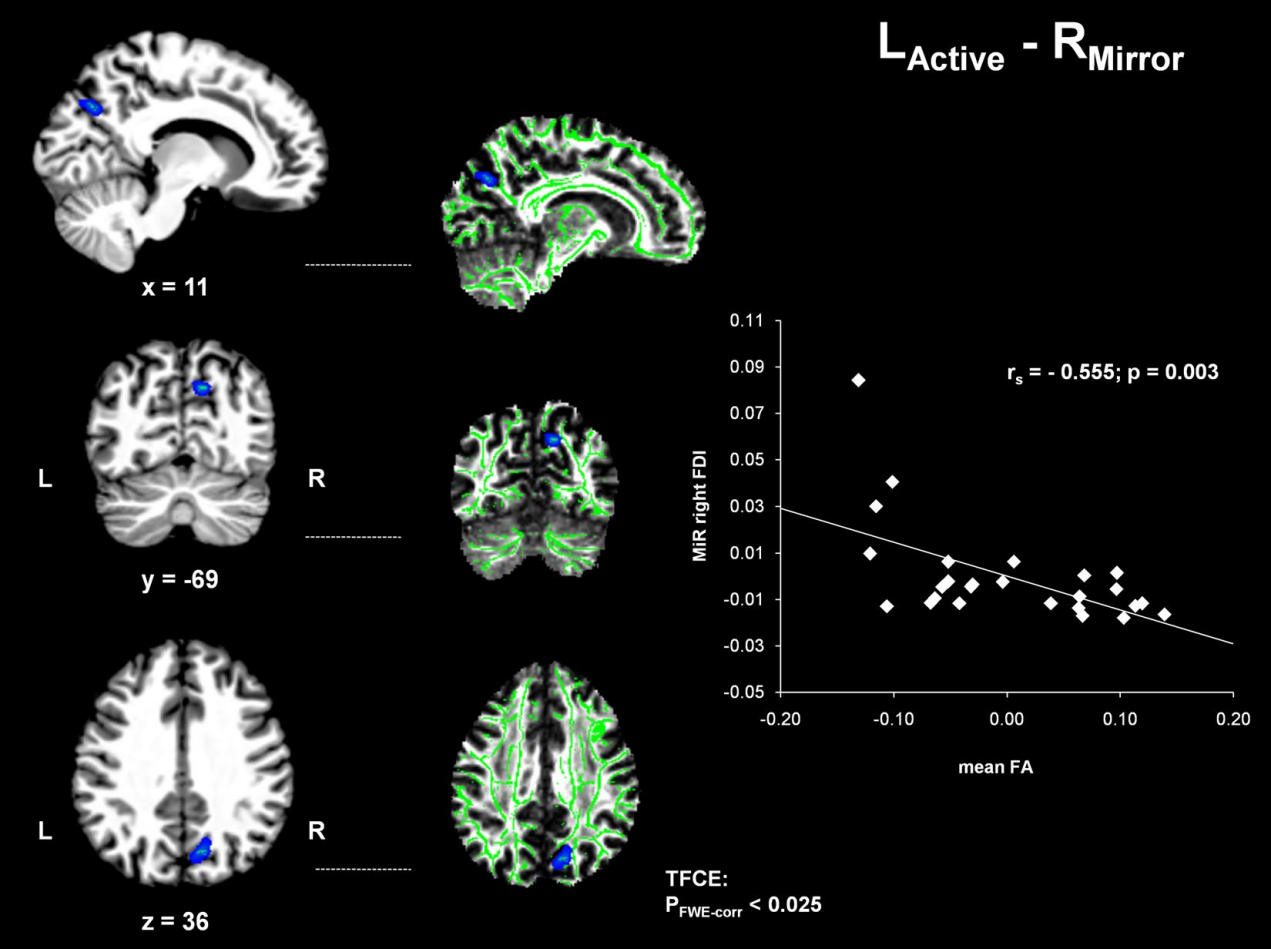
Negative correlation between WM-FA in right PrC and Mirror Recruitment of right FDI (R_Mirror_) during left hand contractions (L_Active_). Significant results of TBSS analysis (p_FWE_ < 0.025) overlaid on T1 image of the most representative subject of the sample in MNI space (left). Corresponding FA map with WM-skeleton (right). Scatterplot displaying the negative correlation between mean FA of significant TBSS-voxels in right PrC and mirror recruitment (MiR) of right FDI.

TBSS analysis showed a negative correlation between MiR of the right FDI with WM FA in right PrC (peak-voxel: 11/−69/36; k = 78; p_FWE_ < 0.025; see Fig. 3). Spearman rank correlation coefficient between extracted mean FA values in native space and MiR of right FDI was r_s_ = 0.555 (p = 0.003; see Fig. 3).

No significant association was found for MiR of left FDI and whole brain WM FA. Again, we tested for possible correlations between WM-FA and each individual force level (20%, 50% & 80% MVC). No significant associations for both FDI were found in these analyses.

## Discussion

In line with previous investigations we observed significant pMA in both dominant and non-dominant hands of healthy male adults during unilateral isometric contractions^12,17–21^. Additionally, we confirmed that pMA increases with increasing force demands of unilateral isometric contractions^12,18–21,32,33^. In general, it seems like there is a connection between the amount of pMA and and the functional characteristics of unilateral motor tasks. Aside from force demands, there are studies showing modulations of pMA in response to repetitive contractions causing central and peripheral fatigue^13,23–25^, increased movement frequency^16^ as well as increased cognitive load during task execution^26^. However, we did not observe differences in the extent of pMA between contractions of the dominant or non-dominant hand. Again, this finding is in accordance to previous studies utilizing similar isometric contractions as compared to our force task^10,12^. Nevertheless, there is also evidence for potential effects of laterality, whereby higher pMA during movements of the non-dominant^16,34^ as well as movements of the dominant hand^35^ have previously been described. Hence, it seems like the underlying characteristics of movement parameters which depend on the task being performed, excepting force requirements, might have an impact on possible side differences in observable pMA^13^.

Here, we provide novel evidence that whole brain correlation analyses by means of VBM and TBSS revealed significant negative correlations between GMV in left ACC and FA in WM of right PrC with the individual tendency to display pMA (MiR) during contractions of the left (non-dominant) hand. No significant associations were found for contractions of the right (dominant) hand.

Medial wall motor structures, i.e. ACC and SMA, generally increase their activity with increasing bimanual task complexity and coordination effort, which could be interpreted as an attempt to decouple both hands and avoid intermanual interference^14,36^. Lesion studies indicated that patients with damaged ACC are unable to perform nonsymmetrical bimanual movements while symmetrical actions are not impaired^37^. In addition, another ischemic lesion study reported overt MM after damage of a large cortical area including the SMA as well as the ACC^38^. Furthermore, unimanual contractions with increasing force demands are generally accompanied by an increase in the activity of the cingulate cortex^39–41^. Anatomically, dense fiber projections between the cingulate cortex and SMA/M1 have been identified in a primate study^42^. Hence, it has been proposed that the ACC exerts a modulatory influence on SMA in order to suppress intrinsically favored symmetrical coordination tendencies (i.e. mirror movements)^15^. In our study, participants with less GMV in left ACC showed a more pronounced tendency for pMA during left-hand contractions. Less GMV in ACC implies a modified cellular basis to generate inhibitory drive on ipsilateral (inactive) primary and secondary motor areas and therefore a facilitated tendency to exhibit pMA during unilateral contractions. This interpretation is supported by another functional investigation of pMA showing an increase in activity of the cingulate cortex during unilateral contractions that might display the inhibitory drive of the ACC on SMA and M1^19^. Decreased inhibition of the ipsilateral (inactive) M1 proceeding from the ipsilateral ACC would accordingly lead to a lower threshold for IHF emerging from the contralateral (active) M1 following the concept of Motor Overflow during strong unimanual contractions^28^. Moreover, it has been shown that cingulate cortex neurons project directly to the spinal cord^42,43^, so that modulatory mechanisms of pMA on a spinal level cannot be ruled out.

The PrC has been identified to be a major hub in the so called “default-brain-network” and seems to be involved in proactive inhibitory processes^44^. Furthermore the PrC in combination with the posterior cingulate cortex has recently been proposed to play a central role inhibiting inappropriate behavior following a “proactive inhibition” model^31,45^. Pathological MM in patients with Parkinson’s disease are associated with an overactivation of bilateral PrC^46^. This finding is interpreted by the authors to be an attempt of the PrC to inhibit abnormal and involuntary behavior (MM). In our study we found a negative correlation between FA in WM of the PrC and MiR during left hand contractions. While the underlying neural basis of FA is debated, it still provides a unique method of assessing the orientation and integrity of neural fibers due to the fact that water diffusion is sensitive to the underlying tissue^47^. A decrease in FA corresponds to an increase in isotropic diffusion of water molecules and might therefore reflect a change in myelination, axon diameter, packing density, or membrane permeability of fiber tracts^47^. Taken together, one can assume that a change in FA indicates that some orientation-dependent aspects regarding the microstructure of the tissue are different^48^. Our results indicate that participants with less FA in WM of contralateral PrC display an increased tendency for pMA during non-dominant hand contractions, which again points to an inhibitory role of PrC during unilateral motor tasks. Therefore, one can assume that the functional neural mechanisms of pathological MM, mainly an overactivation of bilateral PrC^46^, might also be relatable to a structural level under healthy conditions in form of a gradual continuum from healthy to diseased state, reflecting a general inhibitory mechanism of inappropriate behavior (involuntary innervations of resting muscles) that is potentially mediated by transcallosal connections between bilateral PrC during unilateral contractions.

Previous investigations of structural white matter correlates (FA) of pMA were able to show positive associations of excitatory transcallosal fibers connecting bilateral M1 and pMA/IHF during strong contractions of the right (dominant) hand^12,29^. In contrast to facilitatory mechanisms mediated by transcallosal fibers, the CC is also thought to be a critical structure with respect to inhibitory interhemispheric interactions and therefore play a key role in the lateralized transmission of the motor command^8^. Several studies of patients with multiple sclerosis linked an altered microstructure of inhibitory transcallosal motor fibers with poor intermanual coordination during bimanual motor tasks^49,50^, as well as increased MA^51^ and ipsilateral activation of motor areas^52^ during unimanual motor tasks further emphasizing the crucial role of the CC in the inhibition of the contralateral hemisphere. Additionally, there is evidence that the strength of IHI mediated by transcallosal fibers is negatively associated with the amount of pMA in healthy adults during a simple low force unilateral motor task^10^.

At first sight these results seem contradictory but according to the concept of Motor Overflow, the balance of IHI to IHF transmitted through transcallosal fibers changes as a function of applied force during unilateral motor tasks, resulting in bilateral activation of both M1 under high force demands^28^ and accordingly to bilateral muscle innervations and therefore observable pMA.

Nevertheless, it should be mentioned that there is evidence for MA despite agenesis of the CC further supporting a potential role of subcortical as well as spinal mechanisms that are not mediated through transcallosal fiber connections responsible for MA^53^.

Interestingly, in the present study we were not able to replicate these previous associations of transcallosal fiber structure and pMA, neither inhibitory nor excitatory. This might be due to the inherent difference in the methodological approaches applied in these previous studies. Instead of a region of interest (ROI)-driven approach of specifically selected transcallosal fibers we here focused on a whole brain correlation analysis in order to capture additional structural contributors to pMA upstream of primary and secondary motor areas as well as their respective connections. Therefore, it seems likely that a possible relationship between transcallosal fibers and pMA in our study remained below the significance threshold due to the greatly increased number of multiple comparisons that we had to correct for.

Furthermore it should be mentioned that we did not find any relationship of GMV and whole-brain FA with individual force levels examined in this study (20%, 50%, 80% MVC) for both hands. As already mentioned, there seem to be numerous brain structures involved in underlying mechanisms of MA comprising of cortical motor regions, higher order motor/executive areas, subcortical structures and their respective fiber connections^9^. A slight impairment of one of these structures might increase the tendency to exhibit pMA in healthy adults but does not lead to the complete loss of the ability to perform strictly unilateral movements. Therefore it cannot be said that there exists one single key structure responsible for the inhibition or facilitation of involuntary MA^9^. According to this notion it seems reasonable to assume that the relative contribution of these structures responsible for inhibition or facilitation of pMA on each tested force level might differ between individuals and therefore lead to apparent null findings in our whole-brain correlation analyses, possibly also due to a lack of statistical power. Nevertheless, by computing the compound measure of MiR, capturing the tendency to show pMA across all tested force levels we were able to reveal significant negative association at least for contractions of the non-dominant hand. These negative relationships might indicate that the ACC and PrC display general inhibitory influences on pMA during progressively increasing force demands of unilateral contractions that appear to be universally present across neurological healthy individuals.

Lastly, we were not able to show significant associations between brain structure and pMA during contractions of the right (dominant) hand. This result seems interesting regarding the fact, that we did not observe significant differences in the amplitude of pMA between left and right hand contractions. This leads us to hypothesize that the suppression of pMA during dominant versus non-dominant hand contractions might be mediated by differential brain areas and neural networks. This assumption is supported by studies investigating the involvement of the ipsilateral M1 during unilateral movements which show a differential effect of changes in ipsilateral excitability in dependency of the use of the dominant versus non-dominant hand^54,55^. The authors concluded that the left hemisphere (responsible for the control of the dominant hand in right-handers) shows a specialization for complex unimanual movement control which is mediated by functional asymmetries in higher-order motor/executive areas^54^. This asymmetry, possibly due to the higher frequency in everyday use of the dominant hand, could be responsible for the fact that potential associations of brain structure and pMA during right hand contractions remained below threshold in our analyses in contrast to left hand contractions. Nevertheless, one should also take into account that a lack of statistical power in this study might be the reason for non-significant associations of both VBM and TBSS analyses with pMA during right (dominant) hand contractions.

In conclusion, we extend previously described functional associations between ACC/PrC and the suppression of intrinsically favored mirror-symmetrical coordination tendencies to an underlying structural level and complement its inhibitory role to the phenomenon of pMA during non-dominant hand contractions. Taken together we support the notion that the suppression of involuntary MA is not orchestrated by a specific command area or a single underlying brain structure but rather requires a complex interplay and a precisely communicating network comprising of different motor areas^14,56^, higher order motor/executive areas^15,46^, as well as potentially, spinal mechanisms^9,19^ in order to inhibit intrinsically favored mirroring tendencies and eventually restrict the motor command towards the voluntary contracting limb.

## Materials and Methods

### Participants

Twenty-six healthy male adults participated in this study (median (interquartile range (IQR)) age: 23.5 (6.0) years; bodyweight: 74.0 (15.0) kg) which were recruited through public advertisement and from the local Max-Planck Institute participant database. The study was approved by the local ethics-committee of the University of Leipzig. All participants gave their written informed consent to partake in the experiments according to the Declaration of Helsinki, and were remunerated for participation. All participants were right-handed according to the Oldfield handedness inventory^57^ (laterality quotient: 90.0 (19.3)). None of them had any history of playing musical intruments. Additionally, participants were instructed to avoid alcohol and caffeine intake 24hrs prior to testing due to its well-known influences on force production and central nervous system (CNS) functioning^58^.

### Behavioral Experiment

The following descriptions of the experimental setup, as well as methodological and statistical approaches for the analysis of all acquired behavioral data are based on a previous study we conducted^20^. For details, please refer to the respective manuscript.

For our experimental task, all participants were instructed to assume an upright position in a chair with both of their forearms resting comfortably on a table. The active hand was used to control a custom-made force sensor in order to execute an isometric pinch force task (simultaneous contraction of index finger and thumb). During contraction of the active hand, the opposing hand (passive hand) was relaxed, with all participants being instructed to concentrate solely on the active hand. It should be mentioned that at no time did any of our participants receive information regarding pMA to prevent intentional inhibition of involuntarily occurring muscle activity. In advance of testing, a maximum force test was conducted. Particpants exerted individual maximum voluntary contraction force three times (3 s duration for each repetition) with a 1 min resting period in between contractions. To warrant best effort, participants were verbally encouraged following a standardized protocol. For each participant, all trials were averaged and defined as individual maximum voluntary contraction force (MVC). Visual feedback during the pinch force task was provided on a PC using Presentation 16.5 (NeuroBehavioral Systems, Albany, USA). The screen presented a target field and a horizontal bar, with the goal being to move the bar into the target field as quickly and precisely as possible. Target field as well as the force required to reach it were adjusted to individual MVC values, meaning that greater distances required higher levels of force generation. Applied force was displayed on the PC monitor at 60 Hz with a sampling frequency of 800 Hz. The subsequent task utilized a block design comprising of varying force levels (20%, 50%, 80% MVC) relative to each individuals MVC value. For one block, five isometric contractions for one of the force levels. One contraction lasted for 3 s with 3 s rest in between contractions. Since all participants performed 5 contractions per block, this leads a total duration of 30 s per block. Each block was also succeeded by a 30 s resting-period. In total, every participant finished 15 blocks (5 blocks per force level) in pseudo-randomized order to negate order-related effects of muscular fatigue on pMA. The time to complete the task for one hand was 15 min. Following another resting period of 5 min, participants performed the same task for the contralateral hand. It should be mentioned that hand order was also randomized across all subjects.

### EMG Recordings and Analysis

All task-related EMG recordings were monitored on a wireless Desktop Direct Transmission System (NORAXON Inc., Scottsdale, USA). EMG-signals were obtained from bilateral first dorsal interossei muscles (FDI) using bipolar surface electrodes (Ag/AgCl; diameter: 10 mm). To improve comparability across subjects, we standardized inter-electrode distances (20 mm). We also made sure to attach all electrodes parallel to muscle fiber orientation. This setup allowed for us to capture EMG activity over the primary contracting FDI as well as subliminal pMA over the homologous FDI of the relaxed limb. EMG data was recorded with a sample frequency of 1500 Hz, band-pass filtered at 10–500 Hz, input impedance >100 MOhm, Common Mode Rejection Ratio (CMRR) >100 dB and a gain of 500. ProEMG (Motion Lab Systems Inc., Baton Rouge, USA) was used for offline EMG signal processing. First, we rectified all EMG signals and computed the mean EMG activity. This was done by estimating of root mean square values (100 ms) for each burst for all trials and across all recorded muscles. The onset of each EMG burst of the FDI of the active limb was defined as the time point when the mean EMG activity exceeded the baseline EMG-signal (muscles at rest) by 3 standard deviations for a minimal sub period duration of 100 ms. The offset of each EMG burst was defined as the time point when the EMG signal fell below this value for the given time window. Subsequently, we time-locked all EMG recordings from contracting (active) and non-contracting (passive) hands to the on- and offset of the burst of the voluntary contracting muscle. This was done to preserve the temporal relationship between voluntary and involuntary muscular activity. All voluntary EMG amplitudes were normalized with respect to individual MVC values. In accordance to previous investigations of pMA^12,19,20^, pMA amplitudes were expressed as multitudes of the 1000 ms pre-burst baseline EMG signal. This means, that for example, a pMA value of 1 states there was no change compared to the 1000 ms pre-burst baseline signal and a value of 2 states that the involuntarily occurring EMG activity increased by 100% compared to the 1000 ms pre-burst baseline signal. Before any statistical analyses were conducted, we averaged the mean EMG activity for all 25 bursts measured per force level for both volunatry and involuntary FDI.

### Statistical Analysis of Behavioral Data

For all statistical analyses non-parametric methods were used in order to account for the non-normal distribution of the EMG data. For all analyses the statistical threshold was set at p < 0.05 and was appropriately adjusted to correct for multiple comparisons.

Side comparisons of maximal voluntary force during MVC-testing for left and right FDI were calculated using Wilcoxon signed-rank-tests. Within subject comparisons between voluntary mean activities for the left and right FDI of all force levels (FDI_20%_, FDI_50%_, FDI_80%_) were performed using the non-parametric Friedman test of variance by ranks. We calculated additional paired Wilcoxon signed-rank tests for the purpose of post-hoc comparisons between force levels. Accordingly, we used the Dunn-Bonferroni correction to adjust the significance level for 3 pairwise comparisons (FDI_20%_ vs. FDI_50%_, FDI_20%_ vs. FDI_80%_, FDI_50%_ vs. FDI_80%_; α_adjusted_ = 0.0166). We used the same approach to analyze involuntary mean activities of the right and left FDI_pMA_. Here, the significance level of post-hoc Wilcoxon signed-rank tests was Dunn-Bonferroni adjusted for 6 pairwise comparisons (BL vs. FDI_pMA20%_, BL vs.FDI_pMA50%_, BL vs. FDI_pMA80%_, FDI_pMA20%_ vs. FDI_pMA50%_, FDI_pMA20%_ vs. FDI_pMA80%_, FDI_pMA50%_ vs. FDI_pMA80%_; α_adjusted_ = 0.0083).

Furthermore, paired Wilcoxon signed-rank tests were conducted to perform side comparisons of the mean EMG activities of voluntary and involuntary FDI to look for laterality effects. The Pearsons correlation coefficient (r) was used to estimate effect sizes. Therefore, we used z-values of post-hoc performed Wilcoxon signed-rank tests divided by the root of the sample size (√N)^59^.

As a compound measure, capturing the overall tendency for pMA during hand contractions with progressively increasing force demands, we additionally computed the individual slope of involuntary EMG activity across all force levels (FDI_pMA20%_, FDI_pMA50%_, FDI_pMA80%_) for each hand which in a previous investigation has been referred to as mirror recruitment (MiR)^12^. This measure is computed by first averaging all 25 trials of one force level resulting in a mean pMA value for 20%, 50%, 80% MVC for each participant separately. Subsequently the linear slope is computed by these 3 points plus an additional point of 0% MVC/1.0 pMA value, i.e. no mirror activity. Accordingly, the linear slope for each hand and each subject is computed by 4 points (please see Supplementary information - Figures S1 & S2). This compound measure was used for multiple regression analyses with structural and diffusion imaging data. Furthermore, we used the individual pMA values for each tested force level (20%, 50%, 80% MVC) in our multiple regression analysis for structural and diffusion imaging data separately, to look for differential correlates in dependence of applied force during unilateral contractions (see section: Whole-Brain Correlation Analysis).

### MRI Data Acquisition

Participants were scanned on a 3-Tesla Siemens Magnetom PrismaFit scanner using a 32-channel head coil. T1-weighted images were acquired using a MPRAGE (magnetization-prepared rapid acquisition gradient echo) sequence^60^ (voxel size: 1.0 mm isotropic; 176 sagittal slices; Flip Angle: 9°; Field of View (FOV): 256 × 256 mm; repetition time (TR): 2.3 s; echo time (TE): 2.98 ms; inversion time (TI): 900 ms). The acquisition time for the structural scan was approximately 9 minutes. Furthermore, whole brain diffusion weighted imaging (DWI) was acquired with a double spin echo sequence (60 directions; b-value 1000 s/mm²; 88 slices; voxel size: 1.7 mm isotropic, no gap; TR: 6 s; TE: 75 ms; FOV: 220 × 220 mm; GRAPPA acceleration factor = 2; flip angle: 90°) using a mutiband acceleration factor^61^ of 2. Seven volumes without diffusion weighting (b = 0 s/mm²) were acquired, one at the beginning of the sequence and after each block of 10 diffusion weighted images. The acquisition time for the diffusion scan was approximately 10 minutes.

### Structural Data Processing

We performed a VBM (voxel-based morphometry) analysis using CAT12 (Computational Anatomy Toolbox, Jena University Hospital, Departments of Psychiatry and Neurology, Germany; http://www.neuro.uni-jena.de/cat/), a toolbox for SPM12 (Wellcome Trust Centre for Neuroimaging, University College London, London, UK) running under MATLAB (v. R2017b, The MathWorks Inc., Natick, USA). T1-weighted images were modulated and normalized to the MNI 152 (Montreal Neurological Institute) template space and segmented into grey matter (GM), white matter (WM) and cerebrospinal fluid (CSF) using the default approach of combined affine and non-linear registration. Quality of segmented data and sample homogeneity was checked individually for every dataset. Finally, the segmented GM maps were smoothed with a Gaussian kernel of 10 mm full width at half maximum (FWHM).The total intracranial volume (TIV) was included as covariate of no interest in the statistical model (see section Whole-Brain Correlation Analysis).

### Diffusion Data Processing

Diffusion data was analyzed using the FMRIB Software Library (FSL, v. 5.0.9,^62^). Standard preprocessing consisted of correction for susceptibility induced distortions^63^ as well as eddy currents and small head movements^64^. Non-brain tissue was removed using the Brain Extraction Tool (BET,^65^). Finally, local fitting of voxel-wise diffusion tensors was done using the FMRIB Diffusion Toolbox (FDT), resulting in individual fractional anisotropy (FA) maps.

### Whole-Brain Correlation Analysis

We used a Full Factorial Model to analyze the structural MRI data with GROUP as in-between subject factor of no interest. Therefore, we classified the participants into three different groups in accordance to their amount of regular physical exercise. This was motivated by the fact that in our previous work we hypothesized that regular practice of different sports disciplines might affect pMA in specific ways due to long-term neuroplasticity and neural reorganization induced by the respective training regimen^20^. To correct for this possible confound we divided our sample into participants with less than 2 hours per week of combined sports practice (n = 11), endurance type activities of more than 2 hours per week (n = 9) and strength/power type activities of more than 2 hours per week (n = 6). Additionally, we included AGE and TIV as covariates of no interest to correct for different brain sizes. Furthermore, GM maps were threshold-masked with an absolute value of 0.1 to further restrict the analysis to voxels containing GM. Design orthogonality was checked manually. To account for the non-normal distribution of EMG data the TFCE toolbox (v. 133, Jena University Hospital, Departments of Psychiatry and Neurology, Germany) was used for estimation of the multiple regression model based on non-parametric permutation testing using threshold free cluster enhancement (TFCE). Each contrast was estimated with 5000 permutations. The significance threshold was Bonferroni-adjusted to p < 0.025 in order to correct for the analysis of the left and right hand, after family-wise error correction (FWE) for multiple comparisons.

Voxelwise statistical analysis of the FA data was carried out using TBSS (Tract-Based Spatial Statistics,^66^), part of FSL (v. 5.0.9,^67^). All subjects’ FA data were first non-linearly aligned to the most representative subject of the sample and then affine transformed into 1 × 1 × 1 mm MNI standard space. Both transformations were combined before being applied. Next, the mean FA image was created and thresholded at 0.2 to create a mean FA skeleton which represents the centers of all tracts common to the sample. Each subject’s aligned FA data was then projected onto this skeleton and the resulting data was fed into voxelwise cross-subject statistics using a non-parametric permutation testing approach (5000 permutations). Again, a Full Factorial Model with GROUP as in-between subject factor of no interest and AGE as covariate of no interest was used. The statistical threshold at a voxel-level was set to p < 0.025 (Bonferroni-corrected for testing of left and right hand), after FWE correction for multiple comparisons using TFCE^68^. Significant voxels were finally projected back into native space and the individual mean FA values of corresponding voxels were extracted.

Underlying anatomical locations of significant VBM and TBSS results were inferred with atlas tools implemented in FSLview and are displayed in standard MNI space.

### Data Availability

Data, in anonymous format (according to data protection policy in the ethics agreement) is available on reasonable request.

## Electronic supplementary material

Supplementary information - Structural Neural Correlates of Physiological Mirror Activity During Isometric Contractions of Non-Dominant Hand Muscles
