# Supplementary material for: Structural Neural Correlates of Physiological Mirror Activity During Isometric Contractions of Non-Dominant Hand Muscles
Source: Sci Rep. 2018 Jun 15;8:9178. doi: 10.1038/s41598-018-27471-5 (PMC6003937; doi:10.1038/s41598-018-27471-5)
Supplement: Supplementary file 1 — Supplementary information - Structural Neural Correlates of Physiological Mirror Activity During Isometric Contractions of Non-Dominant Hand Muscles [file 41598_2018_27471_MOESM1_ESM.pdf]

Structural Neural Correlates of Physiological Mirror Activity During Isometric Contractions of Non-Dominant Hand Muscles

Tom Maudrich[1,2], Rouven Kenville[1,2], Jöran Lepsien[2], Arno Villringer[2,3,4], Patrick Ragert[1,2]*

[1]Institute for General Kinesiology and Exercise Science, Faculty of Sport Science, University of Leipzig, Leipzig, 04109, Germany
[2] Department of Neurology, Max Planck Institute for Human Cognitive and Brain Sciences, Leipzig, 04103, Germany
[3]Clinic for Cognitive Neurology, University of Leipzig, Leipzig, 04103, Germany
[4]Berlin School of Mind and Brain, Mind and Brain Institute, Berlin, 10099, Germany

*patrick.ragert@uni-leipzig.de

Ethics compliance

The study was approved by the local ethics-committee of the University of Leipzig (ref.-nr. 429-15-16112015). All participants gave their written informed consent to partake in the experiments according to the Declaration of Helsinki, and were remunerated for participation.

Data availability

Data, in anonymous format (according to data protection policy in the ethics agreement) is available on reasonable request.

Supplementary figures

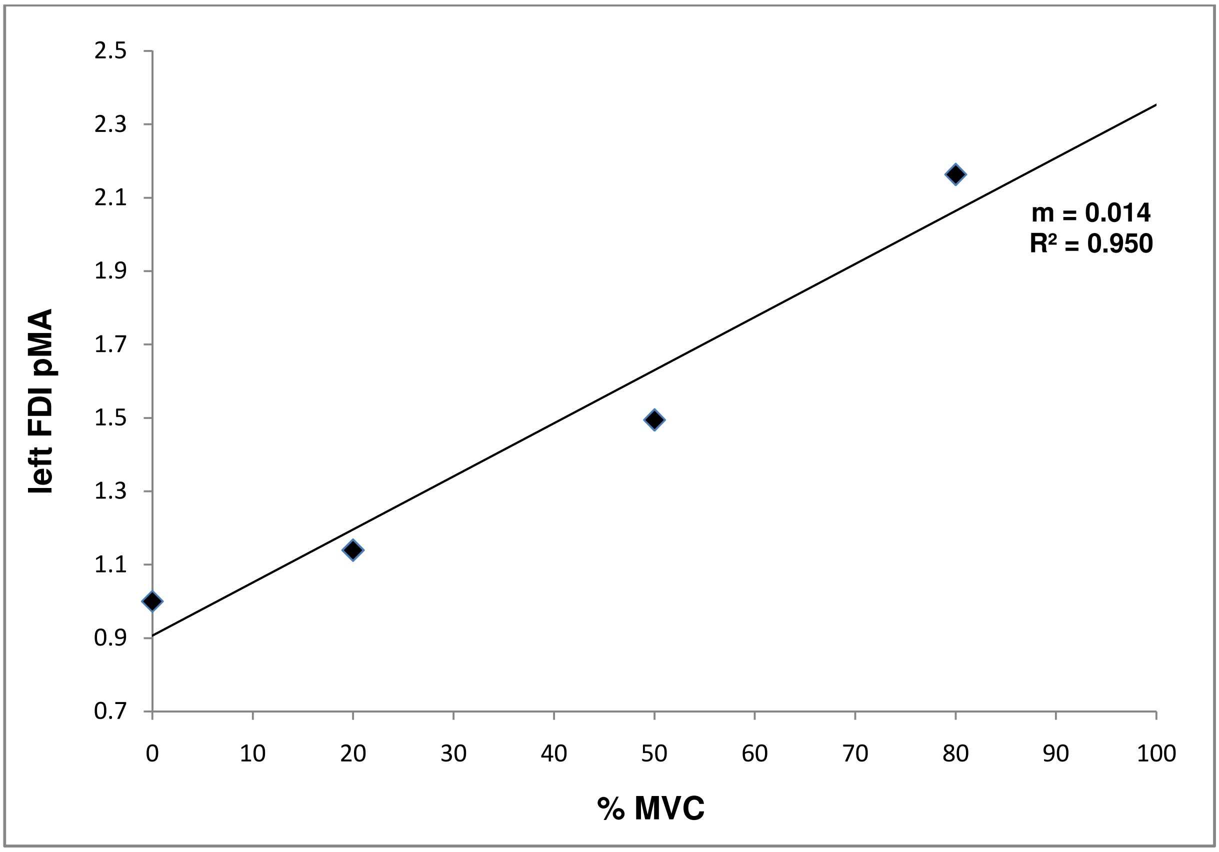

Figure S1. Mean (n = 26) Mirror Recruitment (MiR) of left FDI. pMA values are expressed as multitudes of 1000 ms pre-burst baseline signal, value of 1 = no pMA, value of 2 = 100% increase in pMA compared to baseline activity.

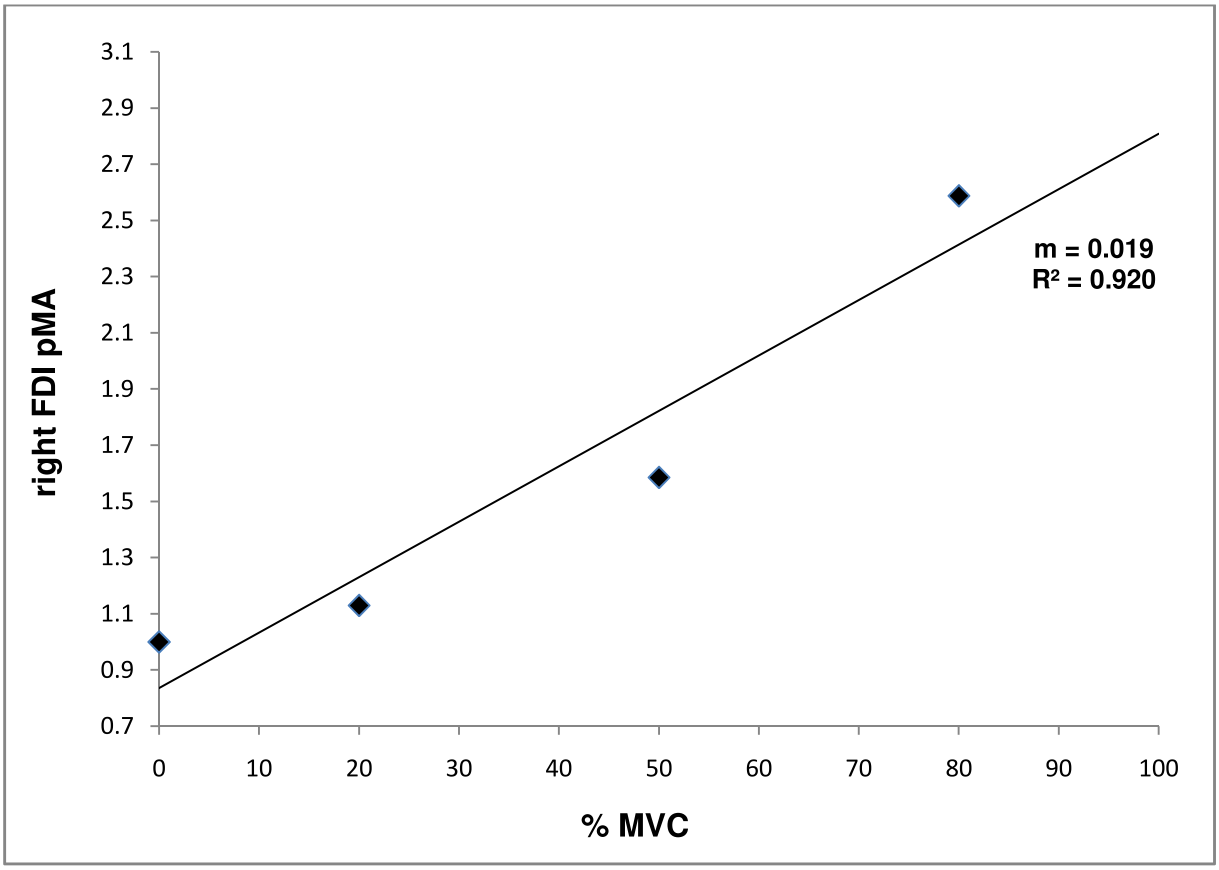

Figure S2. Mean (n = 26) Mirror Recruitment (MiR) of right FDI. pMA values are expressed as multitudes of 1000 ms pre-burst baseline signal, value of 1 = no pMA, value of 2 = 100% increase in pMA compared to baseline activity.

Supplementary results

Table S1. Individual Mirror Recruitment (MiR) values for all participants (P).

P	MiR left FDI	MiR right FDI	P	MiR left FDI	MiR right FDI
**1**	0.0012	0.0068	**14**	0.0012	0.0018
**2**	0.0080	0.0060	**15**	0.0102	0.0172
**3**	0.0395	0.1040	**16**	0.0151	0.0602
**4**	0.0043	0.0081	**17**	0.0003	0.0294
**5**	0.0087	0.0103	**18**	0.0231	0.0174
**6**	0.0081	0.0151	**19**	0.0173	0.0152
**7**	0.0258	0.0026	**20**	0.0382	0.0083
**8**	0.0023	0.0069	**21**	0.0051	0.0260
**9**	0.0049	0.0033	**22**	0.0184	0.0110
**10**	0.0097	0.0081	**23**	0.0027	0.0201
**11**	0.0018	0.0162	**24**	0.0102	0.0080
**12**	0.0094	0.0259	**25**	0.0674	0.0142
**13**	0.0352	0.0499	**26**	0.0081	0.0211

**MEAN**	0.0145	0.0197
**MEDIAN**	0.0091	0.0147
**SD**	0.0157	0.0219
